# Susceptibility of naturally human papillomavirus type 16 major capsid protein L1 variants to vaccines and predictions of future evolutionary trends

**DOI:** 10.1016/j.tvr.2026.200341

**Published:** 2026-04-05

**Authors:** Dongbo Yu, Jiao Wang, Ying Li, Hui Wang, Jieqiong Zhang, Mingming Wang, Haotian Li, Li Zhang, Na Zhu, Xuexin Lu, Shiwen Wang

**Affiliations:** a National Key Laboratory of Intelligent Tracking and Forecasting for Infectious Diseases, National Institute for Viral Disease Control and Prevention, Chinese Center for Disease Control and Prevention, Beijing, 102206, China; b Department of Gynecology, Beijing Obstetrics and Gynecology Hospital, Beijing Maternal and Child Health Care Hospital, Capital Medical University, Beijing, 100026, China

**Keywords:** Human papillomavirus type 16, Pseudovirus, Major capsid protein, Variant, Neutralization

## Abstract

Human papillomavirus type 16 (HPV16) causes over 50% of HPV-related cervical cancers. With the widespread use of virus-like particle (VLP)-based HPV prophylactic vaccines, investigating the differences in viral assembly, infectivity and antigenic properties among different variants is highly important for further eliminating diseases caused by HPV. We analyzed HPV16 L1 protein sequences from the NCBI database and identified 14 representative clusters. Pseudovirus (PsV) formation, infectivity, and susceptibility to vaccines were assessed for these variants, and molecular dynamics (MD) simulations and modified enzyme-linked immunosorbent assays (ELISAs) were ultimately employed to investigate the underlying mechanisms. The sequence identical to P03101 in the UniProt database served as the reference (P16_WT). Among the 13 variants, five presented reduced PsV packaging efficiency to varying degrees, which correlated with diminished L1 protein expression. Compared with P16_WT, the remaining eight variants successfully produced infectious PsVs with high titers and exhibited equivalent infectivity in epithelial cells. Neutralization assays revealed that four variants demonstrated varying levels of decreased susceptibility to vaccines; notably, P16_5 and P16_13 exhibited significantly reduced sensitivity to all three vaccines. MD simulation and modified ELISA indicated that this reduction results from the unstable binding interactions between the L1 variants and the antibodies. Importantly, key residues in these less susceptible variants**,** such as P16_5 and P16_13**,** were under positive selection prior to the commercialization of these vaccines. Our findings demonstrated that a significant proportion of globally circulating HPV16 L1 variants currently display diminished susceptibility to available vaccines. Consequently, ongoing surveillance of HPV variants is imperative.

## 1 Introduction

Cervical cancer is the fourth most common cancer among women globally, with an estimated 660,000 new cases and 348,000 fatalities reported in 2022 by the World Health Organization (WHO) [1]. Persistent infection with high-risk HPV (HR-HPV) strongly contributes to the development of various malignancies, notably cervical cancer, which poses a significant threat to women's health [2,3]. Among them, HPV16 infection is responsible for more than 50% of all cases of cervical cancer [4]. HPV16 L1, the major capsid protein, is relatively conserved and plays a crucial role in viral infectivity, immunogenicity, and vaccine development [5,6]. The L1 gene of this double-stranded DNA virus evolves at a relatively constant rate in the natural state, leading to its diversification into distinct variants [7,8]. Variations in relatively conserved regions alter the structure of the L1 protein, thereby affecting both the efficient assembly of the protein into viral particles and viral infectivity [9,10].

HPV vaccines, which are administered on the basis of virus-like particles (VLPs) composed of L1, are primary preventive measures, with all vaccines containing immunogens against HPV16 [11]. Since the introduction of the initial HPV vaccine in 2006, vaccination rates have consistently increased [12,13]. Research conducted by Chelse Spinner, Silvia de Sanjose, and others has demonstrated a substantial decrease in HPV infection rates postvaccination, leading to a marked reduction in the incidence of HPV-associated cervical cancer [[14], [15], [16], [17]]. However, amino acid mutations in the L1 protein, particularly within the key surface loops (BC, DE, EF, FG, and HI) that constitute the major neutralizing epitopes, escape recognition by human neutralizing antibodies [11]. Ning et al. reported that a single amino acid mutation in the L1 protein affected the efficacy of monoclonal antibodies and guinea pig immune serum against pseudoviruses (PsVs) [18]. Godi et al. illustrated varying sensitivities of different HPV16 lineages to neutralizing antibodies present in nine-valent vaccines [19]. Recent research compiling data from multiple studies has revealed the existence of HPV16 L1 variants and their varied geographic prevalence [20,21]. However, research on the formation, infectivity, and susceptibility of these variants to vaccines is lacking.

This study aims to systematically evaluate the phenotypic characteristics of selected HPV16 L1 variants. Following the selection, we assessed their PsV formation and infectivity in vitro. We then evaluated the sensitivity of these variants to neutralizing antibodies from three common vaccines (Cervarix, Gardasil, and Gardasil-9) and investigated the underlying mechanism. Finally, we performed an evolutionary analysis to determine whether these variants have undergone positive selection. Our findings provide a critical assessment of current vaccine susceptibility against existing HPV16 L1 variants, which is useful for surveillance and next-generation vaccines. The findings of this research will contribute to the understanding of HPV16 genetic diversity, thereby helping us understand evolutionary trends of HPV16 L1 variants.

## 2 Methods

### 2.1 Acquisition and clustering analysis of HPV16 L1 protein sequences

A total of 1437 L1 protein sequences with completeness above 85% were obtained from the NCBI database. Duplicate sequences were merged via CD-HIT, resulting in 228 unique L1 sequences, which were then aligned via the Clustal Omega method via UGENE v49.1. Clustering analysis of the 228 sequences was performed via the maximum likelihood (ML) method using the IQ-TREE software [22]. On the basis of the clustering results, 14 specific clusters were identified, and the consensus sequence from each cluster was used as the final sequence. Notably, all the consensus sequences naturally exist. The sequences that matched the P03101 sequence in the UniProt database were defined as the reference sequence P16_WT. Other variants were numbered as follows: P16_1, P16_2, P16_3, P16_4, P16_5, P16_6, P16_7, P16_8, P16_9, P16_10, P16_11, P16_12, and P16_13.

### 2.2 Synthesis of mutant constructs

The HPV16 L1/L2 expression plasmid and pcDNA3.1-GFP were constructed and maintained in our laboratory. It has been experimentally validated to generate high-titer HPV16 PsVs. The plasmid vector used was a modified pcDNA3.1(+). It contains codon-optimized L1 and L2 genes (*NC_001526*), which are in consensus with p16shell, which was shared by Dr. John T. Schiller [23]. All variant sequences were generated by synthesizing the respective L1 gene and using it to replace the wild-type (WT) L1 sequence in this plasmid. The plasmid map is available from the authors upon request.

### 2.3 Energy calculation via FoldX

We obtained the L1 protein monomer, pentamer, and antigen-antibody complex structure files from the PDB (Protein Data Bank) database and built *L1* monomer and pentamer PDB datasets (PDB ID: 1DZL [24], 3J6R [25],5KEP [26], 5KEQ [26], and 7KZF [27]), as well as an antigen-antibody PDB dataset (PDB ID: 3J8V [28], 3J8W [28],3J8Z [28],6BSP [29],6BT3 [29], and 7CN2 [30]). All PDB files were first optimized for geometry and energy minimized via the RepairPDB command in FoldX 5.0 [31,32]. Next, we corrected some of the mutation sites present in different PDB files on the basis of the *P16_WT* sequence and then introduced multiple mutations to generate different variants via the BuildModel subroutine. The free energy change ΔΔG = ΔG_variant_ - ΔG_WT_ was output by the BuildModel subroutine. We then computed the interaction energy of the mutated complex (ΔG_variant_ and ΔG_WT_) via AnalyseComplex and calculated ΔΔG_bind_ = ΔG_variant_ − ΔG_WT_. In the results section, we report the averages of ΔΔG or ΔΔG_bind_.

### 2.4 Preparation of HPV PsVs

The HPV16 *L1* and *L2* expression vectors and pcDNA3.1-GFP were cotransfected into 293FT cells to produce PsVs. 293FT cells were harvested at 72 h after transfection, lysed in Dulbecco's phosphate-buffered saline (PBS) with magnesium solution containing 0.5% Brij58 (Sigma Aldrich), 0.2% Benzonase (Merck), and 0.2% PlasmidSafe ATP-Dependent DNase (EPICENTRE Biotechnologies), and incubated at 37 °C for 24 h. Afterward, a 5 M NaCl solution was added to the samples to extract the cell lysate. The fluorescence focus units (FFUs) were then measured to determine the titers of the PsVs [33]. All PsVs were purified with a HiScreen Capto Core 700 (Cytiva, America) using AKTA Pure (Cytiva, America). After purification, the PsVs were analyzed via transmission electron microscopy (TEM).

### 2.5 SDS-PAGE and western blotting

SDS-PAGE was performed to detect the expression level of L1. Protein samples were mixed with equal volumes of loading buffer (100 mM Tris-HCl, pH 6.8, 200 mM BME, 4% SDS, 0.2% bromophenol blue and 20% glycerol), heated at 98 °C for 5 min, and then loaded into the wells of a 10% separating gel. Protein samples: Whole-cell lysates were collected 72 h after cotransfection with the plasmid and mixed with loading buffer, and pseudoviruses were lysed with loading buffer.

For Western blotting, separated HPV L1 proteins were transferred to nitrocellulose membranes and blocked with 5% skim milk. After blocking, the membranes were incubated with an HPV16 L1-specific antibody (rabbit polyclonal, Biodragon; B1768; 1:1000 dilution) at room temperature for 2 h and then washed with TBS containing 0.1% Tween-20. The membranes were then incubated with goat anti-rabbit horseradish peroxidase-conjugated antibodies (Abcam; Cambridge, UK; 1:20000 dilution), followed by incubation with enhanced chemiluminescence substrate (PerkinElmer; 0RT2655; 1:1).

### 2.6 Transmission electron microscopy (TEM)

The PsVs in suspension were applied to carbon-coated 200-mesh grids for 5 min, washed once with PBS, and negatively stained for 1 min with filter-sterilized 2% phosphotungstic acid. The grids were examined via an FEI Tecnai F20 transmission electron microscope at an accelerating voltage of 120 kV and photographed at a nominal magnification of × 55,000.

### 2.7 PsV quantification by real-time polymerase chain reaction (qPCR)

Purified PsV preparations were first digested with benzonase (DNase I; Beyotime) to remove unpackaged plasmids. After the 100 μL preparations were inactivated at 95 °C for 5 min, they were subjected to DNA extraction via a DNA/RNA kit (Qiagen) and eluted with 100 μL of double-distilled water (ddH_2_O). To detect the GFP plasmid in the eluate, a Takara probe qPCR kit was used. The amplification of GFP was performed in 0.2 ml 8-strip PCR tubes (Thermo Fisher Scientific) with a total reaction volume of 20 μL. One microliter of each preparation was analyzed in triplicate for each independent experiment. Additionally, GFP plasmids at different concentrations were used to generate a standard curve. The probe and sequences of primers used were as follows: probe: 5′ CTACCCCGACCACATGAAGCAGCACG 3′, F: 5′ GAAGTTCATCTGCACCACCG 3′, R: 5′ TCAAGGACGACGGCAACTAC 3'.

### 2.8 Infection ability analysis

HPV16 PsVs were quantified via qPCR as described above. 293FT cells were seeded into the wells of 96-well plates for 6 h and subsequently infected with initially identical numbers of WT and variant PsVs in a 2-fold dilution series in 10% DMEM. Following a 3-day incubation at 37 °C, the number of fluorophores was counted via an ImmunoSpot reader (CTL, SF6 Flex M2).

### 2.9 Pseudovirus-based neutralization assay (PBNA)

A neutralizing assay was used to detect anti-PsV neutralizing antibodies within the samples. All the serum samples were collected after providing informed and written consent from vaccinated individuals. Briefly, 293FT cells were incubated at 37 °C in the wells of a 96-well plate at a density of 1.5 × 10^4^ cells per well for 6 h. Sera were diluted 2-fold, and PsVs were diluted to 400 fluorescent spots per well. Equal volumes (60 μL) of the PsV diluent and the serially diluted sera were mixed and incubated at 4 °C for 1 h. The negative control was prepared by mixing equal volumes (60 μL) of the PsV diluent and culture medium. Then, 100 μL of these mixtures was added to the designated wells and incubated at 37 °C for 72 h [34]. After incubation, the number of fluorophores was counted with an ImmunoSpot reader. The serum neutralization titers were defined as the 50% maximal inhibitory dilutions and were calculated via the Reed-Muench method [35]. The resulting datasets were statistically analyzed via paired one-way ANOVA via Prism 10.1.2 software.

### 2.10 Molecular dynamics (MD) simulations

On the basis of the structure of the HPV16 PsV with the H16.001 Fab complex (PDB ID: 7CN2) [30], which matches the previous predictions, variant complexes were constructed via FoldX. MD simulations of the complexes were performed via *GROMACS 2024.3* with the CHARMM36m force field. The simulation conditions were set to a constant temperature (310 K), constant pressure (101 kPa), and periodic boundary conditions, and the TIP3P water model was used to simulate the human environment in a neutral 0.15 mol/L NaCl solution. Each system was energy-minimized for 10,000 steps and separately equilibrated with NVT (canonical ensemble, constant number of particles, volume, and temperature) and NPT (isothermal-isobaric ensemble, constant number of particles, pressure, and temperature) ensembles at 310 K and 101 kPa for 0.5 ns and 1 ns, respectively. Then, a production run was performed for 50 ns, in which, every 100 ps, a conformation storage calculation was performed, and the root mean square deviation (RMSD) of the MD simulation results, root mean square fluctuation (RMSF), minimum distance and volume were analyzed and visualized via Xmgrace and VMD [36].

### 2.11 Determination of IgG avidity

The avidity of L1-specific IgG was evaluated via modified ELISA on a subset of samples that were less sensitive to variants [37]. 96-well high-bind plates (Corning) were coated with 50 μL of each HPV VLP (0.1 mg/mL in PBS) per well in duplicate and incubated at 4 °C for 18-20 h. Following washing with 0.05% Tween in PBS, the plates were treated with 1% bovine serum albumin (BSA, Sigma-Aldrich) in PBS for 1 h. Serum samples, selected on the basis of the PBNA results, were appropriately diluted in PBS-1% BSA to achieve an optical density (OD) of 3 ± 0.05%. 100 μL was added to the wells, and the mixture was incubated for 2 h at 37 °C. The plates were subsequently washed with 0.05% Tween in PBS. Each plate included negative and positive controls. The wells were then exposed to 50 μL of 0.5 to 5 M GuHCl per well for 15 min at room temperature under gentle agitation to eliminate low-avidity antibodies while high-avidity antibodies remained bound. Untreated samples and control wells were incubated with PBS alone. After washing with 0.05% Tween in PBS, the immunocomplexes were detected by incubation with goat anti-human IgG-HRP (ZB-2304, ZSGB-BIO, China) for 1 h, followed by additional washing steps. Subsequently, 100 μL of tetramethylbenzidine (TMB) substrate (Solarbio, China) was added, and the mixture was incubated for 15 min at 37 °C. The reaction was stopped by adding 50 μL of stop solution (Solarbio, China). The absorbances at 450 nm and 620 nm were measured via a spectrophotometer (PerkinElmer, USA). The mean OD (OD450 - OD620) of each sample was calculated in duplicate, and the percentage of binding was determined as ((treated OD/untreated OD) × 100). The avidity index (AI) represents the extrapolated molar concentration (M) of GuHCl necessary to reduce the absorbance of the untreated control well by 50%.

### 2.12 Positive selection estimation

The nonsynonymous/synonymous (dN/dS) rate ratio was used to evaluate whether HPV16 L1 has undergone positive selection. The phylogenetic tree constructed above was estimated via Hyphy2.5.8 [38]. Five models were used to investigate whether positive selection could be identified within the 228 HPV16 L1 sequences as follows: αBSREL (Adaptive Branch-Site Random Effects Likelihood), MEME (Mixed Effects Model of Evolution), FUBAR (Fast Unconstrained Bayesian Approximation), FEL (Fixed Effects Likelihood), and SLAC (Single Likelihood Ancestor Counting). This ratio measures the strength and mode of natural selection acting on protein genes, with dN/dS > 1 indicating positive (adaptive or diversifying) selection, and dN/dS ≤ 1 indicating negative (purifying) selection.

## 3 Results

### 3.1 Clustering analysis of HPV16 L1 and L1 variant acquisition

**Fig. 1:**
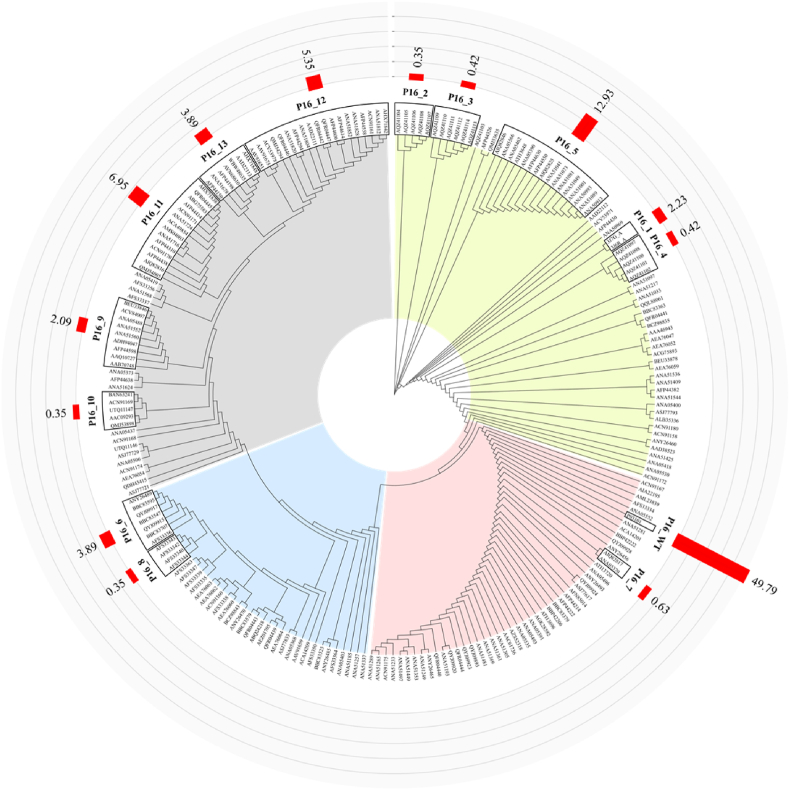
Phylogenetic tree clustering analysis of 228 L1 protein sequences. The four different colored sectors represent the four major clusters. The black borders on the outer edges highlight the 14 selected groups, with their consensus sequences serving as the final variant protein sequence for each cluster. These consensus sequences naturally exist. The red bars represent the prevalence of each cluster.

**Fig. 2:**
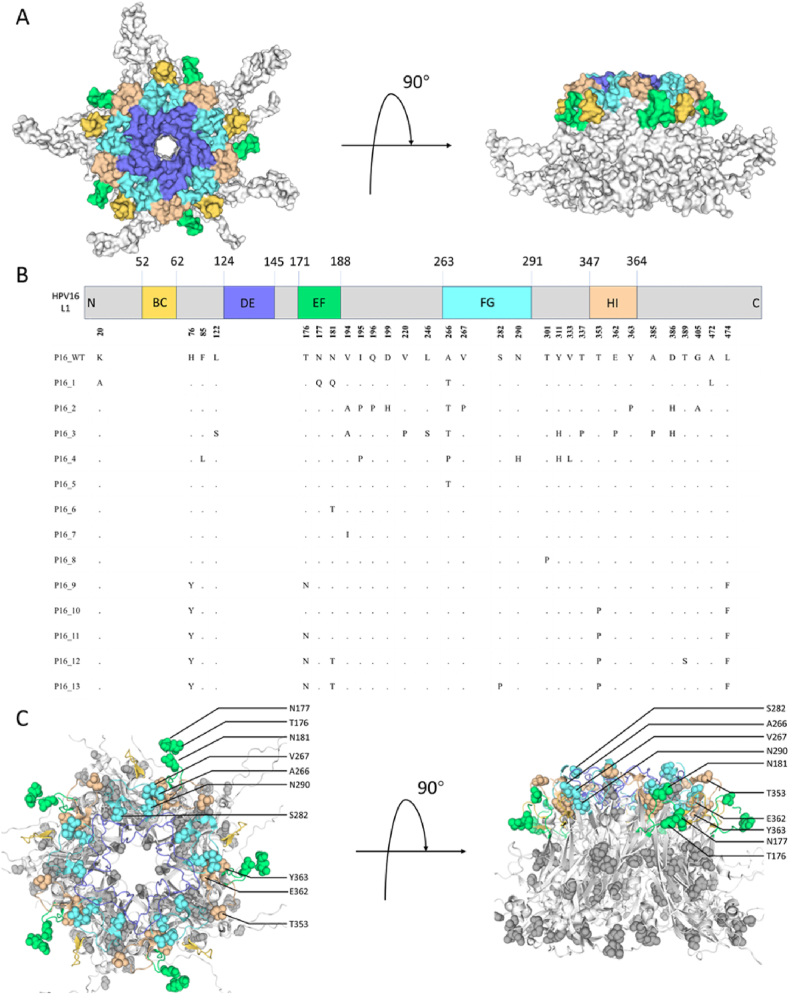
Mutation sites of L1 variants. A, L1 pentamer structure, different colors represent surface-exposed loop structures, BC loop shown in yellow, DE in blue, EF in green, FG in cyan, HI in wheat. B, Mutation sites in 13 variants compared to WT are distributed at the corresponding positions of L1. The dots indicates that there is no mutation at that position. C, The 30 mutation sites from the 13 variants were mapped onto the corresponding positions on the L1 pentamer structure. All 30 residues are shown as atom spheres colored in relative colors and 10 residues located in surface loops were labeled.

After clustering, 228 nonrepetitive L1 protein sequences predominantly formed four major clusters, which were further subdivided into numerous smaller clusters. These clusters varied in prevalence from 0.07% to 49.8%. We selected 14 clusters (Fig. 1) and assigned them corresponding identifiers. The consensus sequence of each cluster was designated as its representative protein sequence, and all the consensus sequences naturally exist. The cluster is consistent with the UniProt P03101 sequence, which was labeled the reference sequence (P16_WT) and had the highest prevalence at 49.8%. The prevalence of the remaining 13 variants is displayed in Fig. 1. These 13 variants are distributed across different branches surrounding P16_WT, indicating broad sequence diversity and evolutionary divergence within the analyzed population, despite the high degree of conservation observed relative to the reference sequence. Five loops which are important epitopes were colored in relative colors (Fig. 2A). Compared with P16_WT, 30 mutation sites among these 13 variants were presented and were distributed throughout L1 (Fig. 2B). Mutations were primarily concentrated in the EF, FG, and HI loops, among the five loops (Fig. 2B). All the 30 residues were mapped onto the corresponding positions on the L1 capsomere structure and 10 residues which are located in the loops were labeled (Fig. 2C). The remaining 20 sites, predominantly located within relatively conserved structural regions, were similarly mapped onto their corresponding positions within the L1 pentamer structure (Supplementary Fig. 1).

### 3.2 Predicted effects of L1 variants on their free energy and antigen-antibody binding energy

**Fig. 3:**
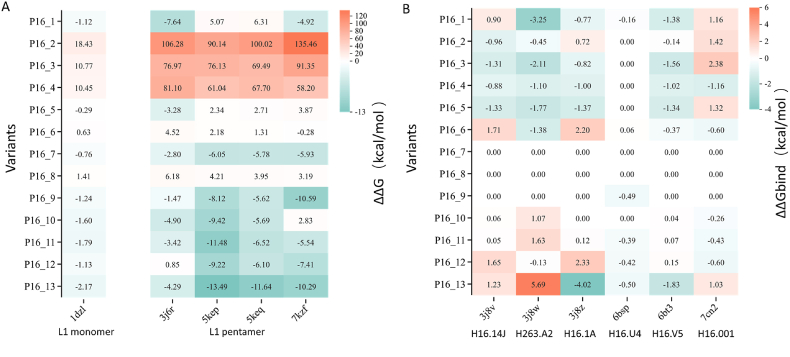
Heatmap of the predicted ΔΔ G and ΔΔG_bind_ values via FoldX for variants from different s**tructures.** The vertical axis displays the different variants. The horizontal axis represents the PDB IDs for either the L1 monomer/pentamer structures (A) or the antibody-L1 complex structures (B). The legend under the PDB ID represents the L1 monomer and pentamer structures in panel A, while in panel B, it represents the identifiers of the respective monoclonal antibodies (mAbs). The intensity of the color corresponds to the magnitude of the correlation coefficient.

To assess the impact of the 13 variants on PsV formation and vaccine-induced neutralizing antibody recognition, we predicted changes in free energy and binding energy (Fig. 3). For L1 monomers and pentamers (Fig. 3A), predictions indicate that variants P16_2, P16_3, P16_4, and P16_8 exhibit increased free energy (ΔΔG >0) compared with P16_WT. Moreover, most predictions for P16_5 and P16_6 also show increased free energy (ΔΔG >0). These results indicate that the above variants destabilize the L1 monomer and/or pentamer. In contrast, the remaining variants generally show decreased free energy (ΔΔG <0), implying enhanced protein stability.

Vaccine-induced protection against HPV16 is mediated primarily by neutralizing antibodies. Amino acid substitutions that alter the antigen-antibody interface can modulate binding affinity, potentially impacting the efficacy of such neutralization. We assessed the impact of the variants on the binding energy (Fig. 3B). The results indicate that variants P16_7, P16_8, P16_9, and P16_10 exhibit no significant change in binding energy (ΔΔG_bind_ = 0), suggesting that these mutations do not affect L1-antibody interactions or that there are no epitopes recognized by the antibody, thereby maintaining vaccine efficacy against these variants. Conversely, variants P16_1, P16_2, P16_3, P16_4, and P16_5 show decreased binding energy (ΔΔG_bind_ < 0), implying tighter L1-antibody binding, potentially enhancing vaccine efficacy. For P16_6, P16_11, P16_12, and P16_13, the binding energy changes are balanced, aligning with the expected complexity and diversity of antibodies in serum. The construction of the antigen-antibody PDB dataset was intended to simulate this complexity.

### 3.3 Effects of L1 variants on virus particle formation and infectivity

**Fig. 4:**
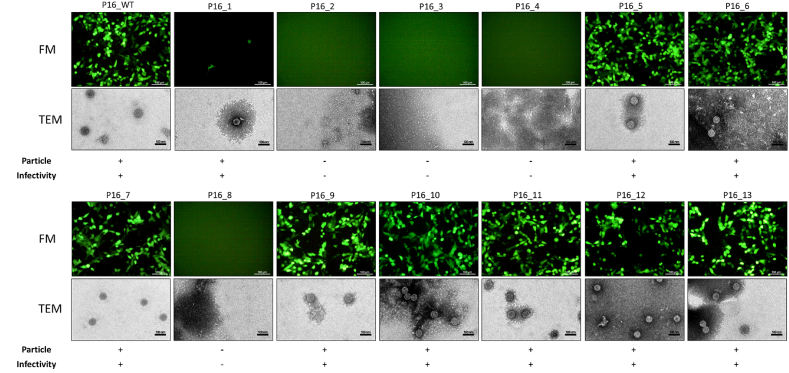
The impact of variants on pseudovirus formation. Negative-stain transmission electron microscopy (TEM) and fluorescence microscopy (FM) were used to detect pseudovirus particle formation and infectivity. Scale bar in TEM view, 100 nm; scale bar in FM view, 100 μm. One representative image from three biological replicates is shown for each group.

**Fig. 5:**
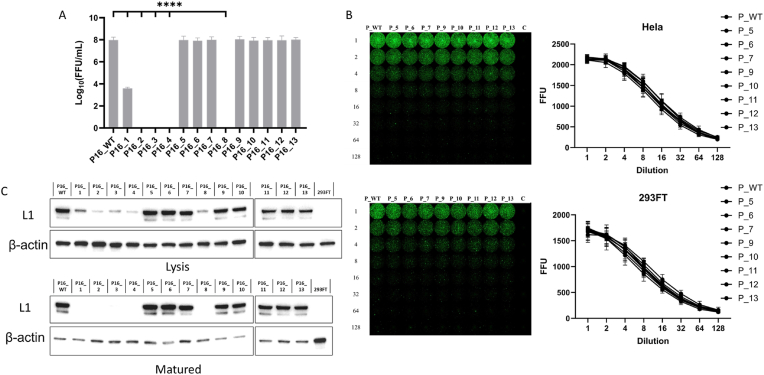
Pseudovirus titer and ability to infect cells. A. Titers of different pseudoviruses. B. Infection efficiency of different pseudoviruses in HeLa and 293FT cells. Pseudoviruses with equal genome copy numbers were subjected to 2-fold serial dilution before infection. C. Western blotting of the L1 protein in different pseudovirus variants before and after maturation via an anti-L1 antibody. "Lysis" indicates whole-cell lysates prepared with loading buffer prior to maturation; "Matured" indicates lysates of pseudoviruses that were treated with 0.17 vol of 5 M NaCl after 24 h of maturation, followed by lysis with loading buffer. *, *P* < 0.05; **, *P* < 0.01; ***, *P* < 0.001; ****, *P* < 0.0001; paired one-way ANOVA.

We constructed PsVs for 14 L1 clusters. The results indicated that four variants, P16_2, P16_3, P16_4, and P16_8, failed to form infectious PsV particles, aligning with FoldX predictions. These variants resulted in no detectable PsV titers, and no viral particles were visible via transmission electron microscopy (Fig. 4). The other nine variants successfully formed infectious PsV particles (Fig. 4). Although P16_1 formed PsVs, its titer was extremely low (Fig. 5A). P16_WT and the remaining eight variants produced high-titer PsV particles, with no significant differences in titer (Fig. 5A). Transmission electron microscopy revealed no notable structural or size differences (Fig. 4). To investigate potential differences in epithelial cell infectivity among these variants, HeLa and 293FT cells were infected with equal numbers of copies of the corresponding PsV. Compared with P16_WT, the eight variants demonstrated no significant differences in their capacity to infect epithelial cells (Fig. 5B).

A Western blot analysis (Fig. 5C) was conducted to investigate why the variants failed to form PsVs. All 13 variants expressed the protein at different levels before PsV maturation. The eight variants that successfully formed high-titer PsVs presented L1 protein expression levels comparable to those of P16_WT. In contrast, the four nonforming variants presented the lowest expression levels, with P16_1 displaying an intermediate level. After maturation, the L1 protein was detected only in the eight successful variants and P16_WT, while it was undetectable in the other variants. These findings suggest that these five variants may significantly reduce L1 expression or stability and ultimately lead to the failure of virus particle formation.

### 3.4 Analysis of the neutralizing abilities of HPV16 L1 variants to vaccine-induced antibodies

**Fig. 6:**
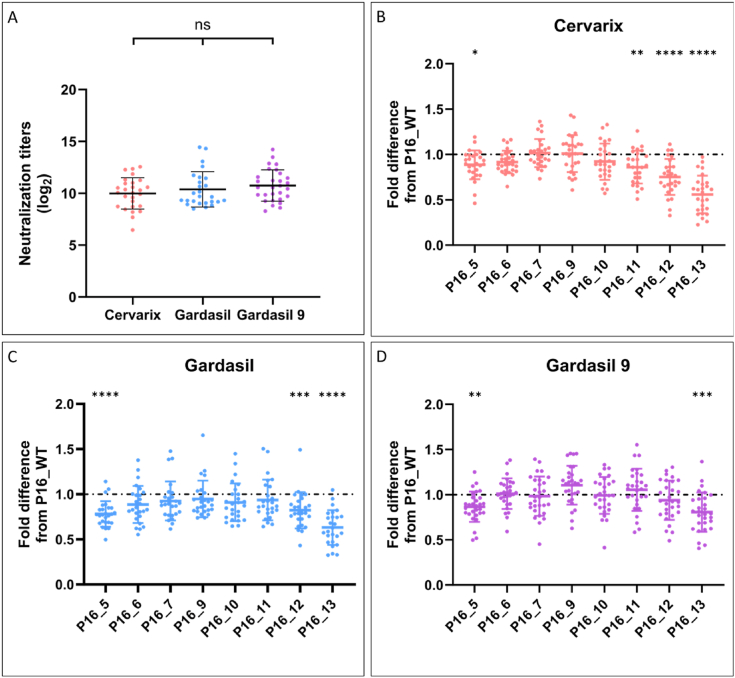
Differences in the 50% maximal inhibitory dilution (ID50) between variant pseudovirions (PsVs) and the reference PsVs against immunized fe**male sera.** A. Neutralizing titers against P16_WT for the three vaccines. B. C. and D represent the fold changes in the ID50 values of the reference pseudovirions for Cervarix (n = 27), Gardasil (n = 26), and Gardasil 9 (n = 29)-immunized sera. Each point represents the fold difference in ID50 between a variant and the P16_WT PsVs. *, *P* < 0.05; **, *P* < 0.01; ***, *P* < 0.001; ****, *P* < 0.0001; paired one-way ANOVA.

The susceptibility of P16_WT and eight variants to neutralizing antibodies induced by the Cervarix (n = 27), Gardasil (n = 26), and Gardasil-9 (n = 29) vaccines was assessed (Fig. 6). Serum samples, primarily collected within a month post-vaccination following a three-dose regimen from individuals under 50 years of age, were analyzed. First, we evaluated the neutralizing efficacy of the three vaccines against P16_WT (Fig. 6A). Although slight fluctuations in neutralizing titers were observed across the vaccine cohorts, the differences were not statistically significant.

We subsequently examined the susceptibility of distinct variants to the three vaccines individually. The efficacy of Cervarix (Fig. 6B) was notably diminished against P16_5, P16_11, P16_12, and P16_13, with the most pronounced reduction in P16_12 and P16_13, where the neutralizing capacity was approximately 70% and 50% that against P16_WT, respectively. Similarly, Gardasil also showed decreased efficacy against P16_5, P16_12, and P16_13, with the lowest neutralization, approximately 60% of P16_WT, observed against P16_13 (Fig. 6C). Notably, Gardasil compensated for the reduced neutralizing ability against P16_11 observed with Cervarix. For Gardasil-9, only P16_5 and P16_13 exhibited reduced sensitivity, maintaining approximately 80% neutralization compared with P16_WT (Fig. 6D). Gardasil-9 enhanced the neutralization of P16_13 and restored protection against P16_12, while its efficacy against P16_5 remained comparable to that of the other vaccines. Interestingly, despite FoldX predicting a decrease in binding energy for P16_5 with antibodies (ΔΔG <0, indicating more stable binding), all three vaccines exhibited diminished neutralizing activity against this variant. However, the FoldX predictions for P16_7, P16_9, and P16_10 remained consistent with the experimental outcomes.

### 3.5 MD simulation and antibody avidity analysis

**Fig. 7:**
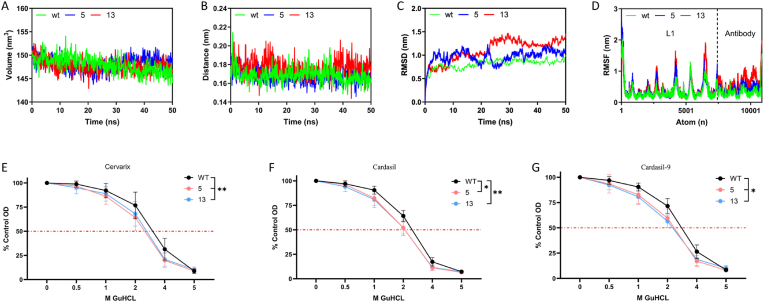
MD simulation results and antibody avidity analysis. A. Minimum distance between L1 and antibody interactions during the MD simulations. B. Volume of the L1-antibody complex. C. D. RMSD, and RMSF of the L1-antibody interactions. E. F. G. The avidity of L1-specific IgG in the serum of individuals receiving different vaccinations was evaluated via a modified ELISA.

The distinct neutralization properties of the P16_5 and P16_13 variants prompted further investigation into their structural dynamics when complexed with antibodies. MD simulations were performed for P16_WT, P16_5, and P16_13 in complex with the monoclonal antibody H16.001 Fab (PDB ID: 7CN2), which aligns with the binding energy predictions (Fig. 3B). The parameters examined included the minimum distance between the antigen and the antibody, the complex volume, the root-mean-square deviation (RMSD), and the root-mean-square fluctuation (RMSF). The variants did not alter the antigen-antibody complex volume (Fig. 7A), and the minimum distance shifted slightly, with P16_13 being marginally larger than P16_WT (Fig. 7B). However, P16_5 and P16_13 presented greater RMSD fluctuations than P16_WT did (Fig. 7C), suggesting reduced binding stability. The RMSF results corroborate this perspective, showing increased fluctuations in the antibody bound to variants, which leads to unstable binding (Fig. 7D).

To corroborate the findings of the dynamic simulations, a modified ELISA was utilized in this investigation. The outcomes demonstrated that, in comparison with P16_WT, variants P16_5 and P16_13 presented diminished avidity to antibodies present in the sera induced by all three vaccines (Fig. 7E, F, G). An analysis of key amino acids and the number of hydrogen bonds between L1 and the antibodies was conducted, which further confirmed these findings (Supplementary Fig. 2).

### 3.6 Evolutionary analysis of HPV16 L1: identification of positive selection

To investigate the potential evolutionary trends of HPV16 L1, we performed a selection pressure analysis by calculating the dN/dS ratio. The results from the αBSREL model indicated that 3.1% of the branches had undergone positive selection. Among these variants, amino acid sites N181 and A266, which are included in variants P16_13 and P16_5, respectively, underwent positive selection (Table 1).

**Table 1:** Evolutionary analysis using the dN/dS rate ratio.

	dN/dS > 1			
Sites	MEME	FUBAR	FEL	SLAC
9	+	+	+	-
113	+	+	+	-
178	+	+	-	-
181	+	+	+	+
195	+	+	-	-
266	+	+	+	+
285	+	+	-	-
389	+	+	-	-
423	+	+	+	-
492	+	-	+	-

+: dN/dS > 1, -: dN/dS ≤ 1.

**NOTE:** The four models used to estimate positive selection are as follows: MEME (Mixed Effects Model of Evolution), FUBAR (Fast Unconstrained Bayesian Approximation), FEL (Fixed Effects Likelihood), and SLAC (Single Likelihood Ancestor Counting).

These findings suggest that HPV16 L1 has been subject to spontaneous positive selection as part of its natural diversification process. Since these variants predate the vaccine era (P16_5 and P16_13 were isolated in 1994 and 1996 respectively), the identified mutational profiles reflect an evolutionary trajectory that developed independently of vaccine-induced selective pressure. Combined with the results of their reduced sensitivity, these findings indicate that the observed amino acid substitutions are the result of long-term viral adaptation and that the L1 gene may continue to diverge along these naturally established variants, such as those represented by P16_5 and P16_13.

## 4 Discussion

The L1 protein is the primary structural component of the HPV16 capsid, and its sequence conservation is fundamental for maintaining structural integrity and host cell infectivity. Through a systematic evaluation of natural variants, this study revealed that the inherent diversity of HPV16 L1, shaped over long-term evolution, modulates both capsid assembly efficiency and neutralization sensitivity. These findings provide critical insights into the natural evolutionary trends and diversification of the biological characteristics of HPV16.

Among the 13 variants analyzed, four (P16_2, P16_3, P16_4, and P16_8) failed to form PsVs in 293FT cells, a commonly used PsV preparation system. This may be due to mutations affecting L1 protein expression and/or stability [18]. For the nine variants that were successfully assembled, transmission electron microscopy confirmed that their morphological integrity remained consistent with that of P16_WT, and most maintained infectivity levels comparable to those of the reference sequence, with the notable exception of P16_1, which presented significantly lower titers. The sensitivity to vaccines varied across variants: P16_11 exhibited reduced sensitivity exclusively to the bivalent vaccine (Cervarix), whereas P16_12 displayed resistance to both bivalent and quadrivalent (Gardasil) vaccines. Most notably, P16_5 and P16_13 exhibited significantly reduced neutralization sensitivity across all three major vaccines, and the mechanism is due to a reduced avidity of L1-antibody binding, which leads to disintegration of the complex. Given that these two variants alone represent approximately 17% of the global L1 prevalence, their widespread distribution is of particular epidemiological challenge (Fig. 1). These findings highlight that targeted, continuous surveillance of HPV16 L1 variants is essential for disease prevention and the refinement of public health strategies.

HPV vaccination is a pivotal measure for curbing HPV transmission and prevalence [39]. The study revealed diminished efficacy of the three vaccines against P16_5 and P16_13, while higher-valent vaccines exhibited enhanced protection against variants. This phenomenon may be attributed to the presence of neutralizing antibodies targeting other HPV types in Gardasil-9, potentially conferring cross-protection against HPV16 [40,41].

HPV16 PsVs are assembled of L1 and L2 proteins. Our primary focus was to assess the influence of L1 variants on their formation, infectivity and susceptibility to current mainstream L1 VLP vaccines. There is currently no evidence indicating cross-reactivity between L1 VLP vaccines and L2 or that L2 sequences impact vaccine recognition by L1. Consequently, we generated PsVs using the same L2 sequence (NC_001526). Several variants identified in this study, primarily those isolated from cervical cancer (Supplementary Table 1), failed to generate pseudoviruses (PsVs), though it remains unclear whether this failure is attributable to viral integration [42]. Although the inability to form virions effectively blocks viral transmission, whether the sequences isolated from clinical samples accurately represent the actually circulating variants remains a critical unknown. Our results indicate that the four variants that cannot form PsVs are caused by the low expression or stability of L1. However, completely ruling out the potential impact of the L2 protein sequence and the expression system is also not possible, which is a hypothesis that warrants further investigation.

These data collectively demonstrate that the sensitivity of HPV16 L1 variants to vaccine-induced neutralizing antibodies differs. Molecular dynamics simulations and validation experiments suggest that variants may destabilize L1-antibody binding, diminishing the ability of antibodies to neutralize HPV16. By comparing the mutational profiles of variants from P16_9 to P16_13, we identified a consistent mutational signature. This signature is characterized by the co-occurrence of mutations ranging from H76Y to L474F, including T176N and/or T353P. The diminished sensitivity of these variants to the vaccine is not attributed to the mutation of individual key amino acid sites but rather to the cumulative effect of these sites [19]. Current research suggests that variants may continuously accumulate beneficial mutations to facilitate their evolution [18]. The possibility cannot be excluded that the ongoing evolution of HPV16 may further challenge the efficacy of existing vaccines. Monitoring HPV16 L1 variants and developing broad-spectrum vaccines are essential for effective HPV prevention and control. Consequently, the development of effective, broadly protective vaccines and their evaluation should remain a priority.

## CRediT authorship contribution statement

**Dongbo Yu:** Writing – review & editing, Writing – original draft, Validation, Methodology, Investigation, Formal analysis, Data curation, Conceptualization. **Jiao Wang:** Writing – review & editing, Investigation, Data curation. **Ying Li:** Methodology, Formal analysis, Data curation. **Hui Wang:** Writing – review & editing, Resources. **Jieqiong Zhang:** Writing – review & editing, Resources. **Mingming Wang:** Writing – review & editing, Resources. **Haotian Li:** Writing – review & editing, Formal analysis. **Li Zhang:** Writing – review & editing, Resources. **Na Zhu:** Writing – review & editing, Validation, Supervision, Project administration, Methodology, Funding acquisition. **Xuexin Lu:** Writing – review & editing, Validation, Supervision, Methodology, Conceptualization. **Shiwen Wang:** Writing – review & editing, Supervision, Project administration, Conceptualization.

## Ethics statement

All the serum samples were collected after providing informed and written consent and were approved by the Ethics Committee of Beijing Obstetrics and Gynecology Hospital, Capital Medical University.

## Funding

This work was supported in part by grants from the Fund of the National Key Laboratory of Intelligent Tracking and Forecasting for Infectious Diseases (ZDWNLJS25-37), the 10.13039/501100001809National Natural Science Foundation of China (82072296) and the 10.13039/501100012166National Key Research and Development Program of China (2022YFC2604101). The funders had no role in the study design, data collection and analysis, decision to publish, or preparation of the manuscript.

## Declaration of competing interest

The authors declare that they have no known competing financial interests or personal relationships that could have appeared to influence the work reported in this paper.

## Data Availability

Data will be made available on request.
