# Supplementary material for: Susceptibility of naturally human papillomavirus type 16 major capsid protein L1 variants to vaccines and predictions of future evolutionary trends
Source: Tumour Virus Res. 2026 Apr 5;21:200341. doi: 10.1016/j.tvr.2026.200341 (PMC13091289; doi:10.1016/j.tvr.2026.200341)
Supplement: Multimedia component 1 [file mmc1.pptx]

## Slide 1
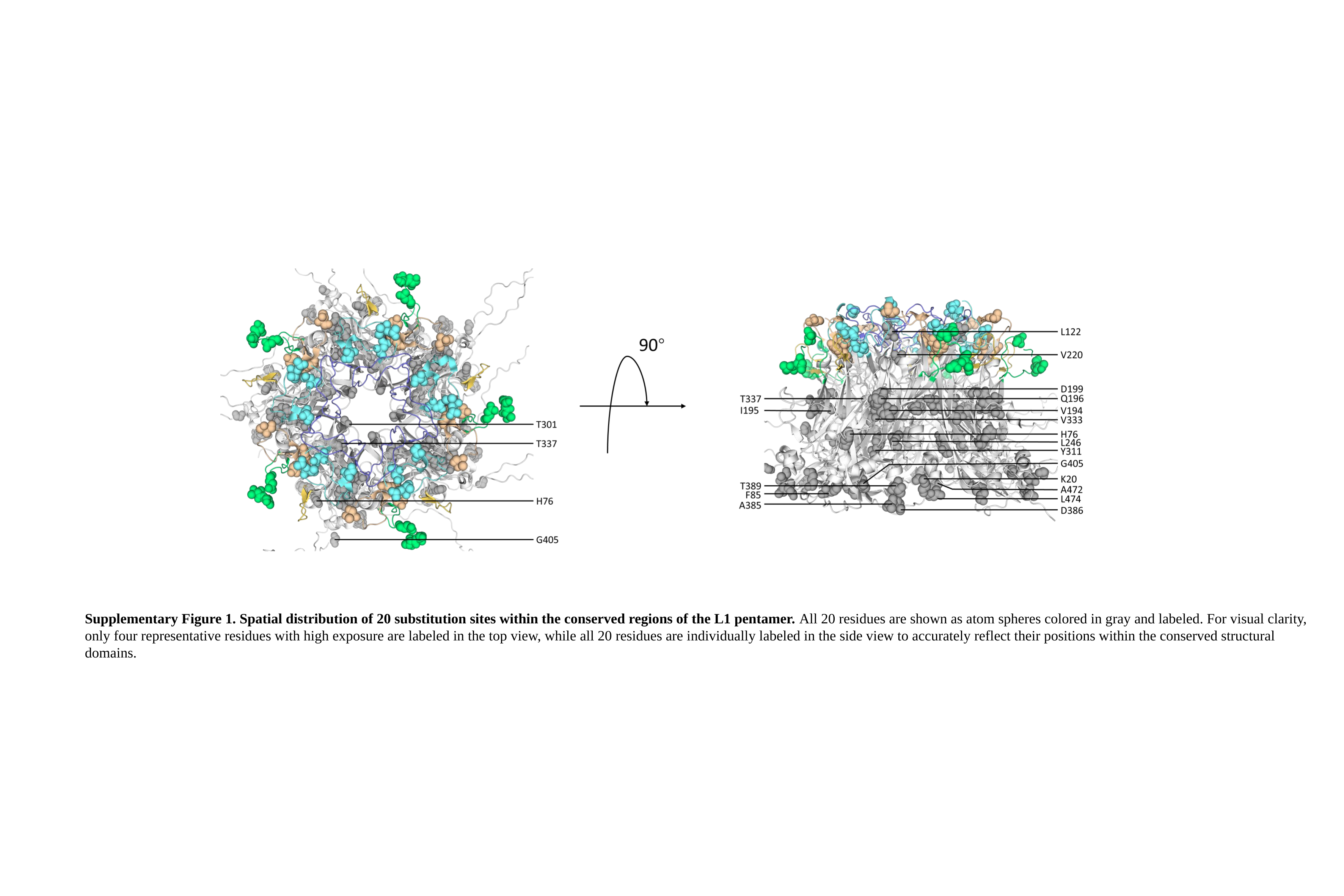

Supplementary Figure 1. Spatial distribution of 20 substitution sites within the conserved regions of the L1 pentamer. All 20 residues are shown as atom spheres colored in gray and labeled. For visual clarity, only four representative residues with high exposure are labeled in the top view, while all 20 residues are individually labeled in the side view to accurately reflect their positions within the conserved structural domains.

## Slide 2
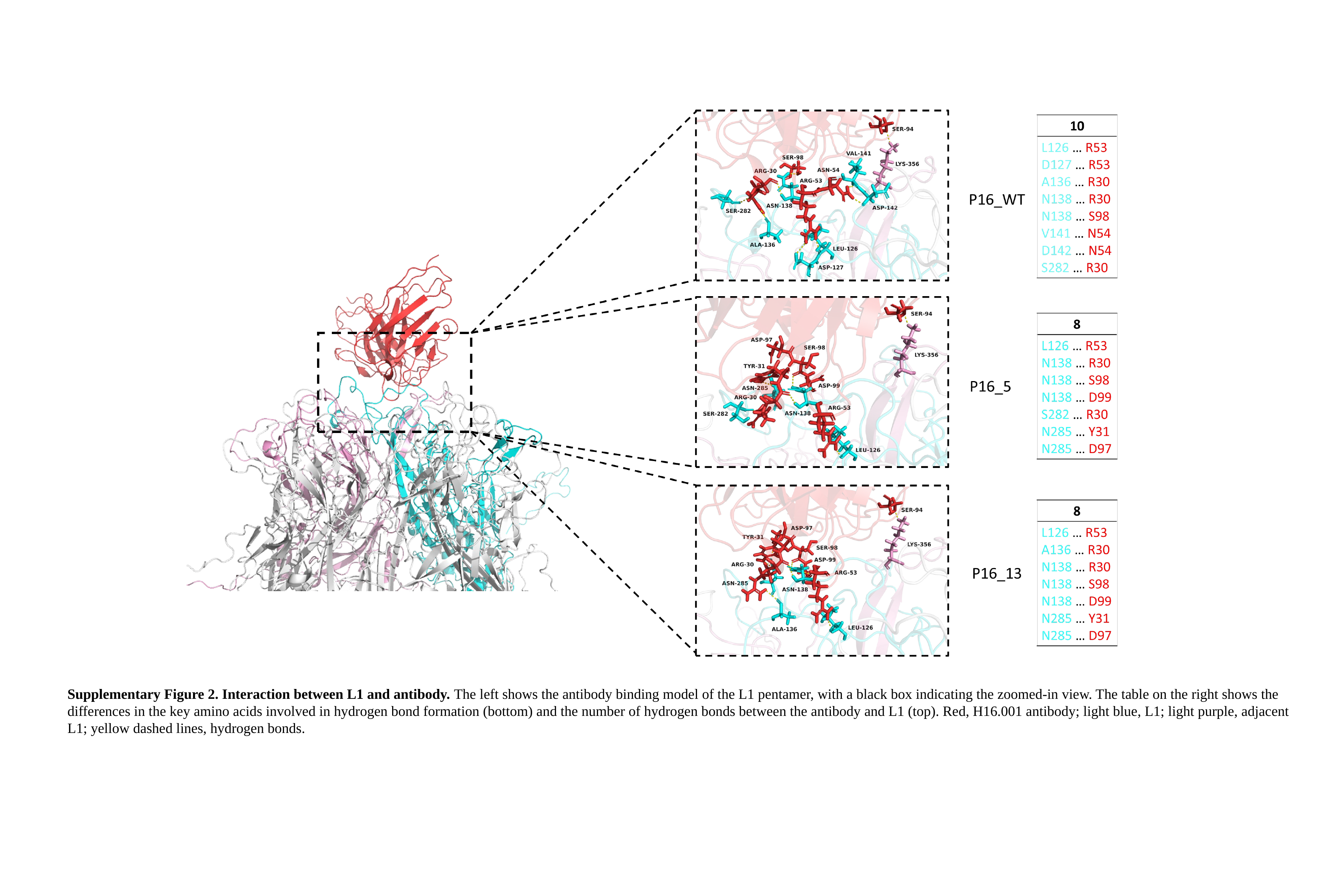

Supplementary Figure 2. Interaction between L1 and antibody. The left shows the antibody binding model of the L1 pentamer, with a black box indicating the zoomed-in view. The table on the right shows the differences in the key amino acids involved in hydrogen bond formation (bottom) and the number of hydrogen bonds between the antibody and L1 (top). Red, H16.001 antibody; light blue, L1; light purple, adjacent L1; yellow dashed lines, hydrogen bonds.
