# Supplementary material for: Susceptibility of naturally human papillomavirus type 16 major capsid protein L1 variants to vaccines and predictions of future evolutionary trends
Source: Tumour Virus Res. 2026 Apr 5;21:200341. doi: 10.1016/j.tvr.2026.200341 (PMC13091289; doi:10.1016/j.tvr.2026.200341)
Supplement: Multimedia component 2 [file mmc2.docx]

**Supplementary Table 1.** **Clinical Sources of Isolated Viral Variants**

| **Isolate sources** | | | | | | | | |
| --- | --- | --- | --- | --- | --- | --- | --- | --- |
| Variants | Cervical scrapes | Cervical cancer | | LSIL | HSIL | Unspecified | Unknown | Total |
| P16_WT | 252 | 40 | 8 | | 3 | 0 | 410 | 713 |
| P16_1 | 0 | 0 | 0 | | 0 | 0 | 20 | 20 |
| P16_2 | 0 | 0 | 0 | | 0 | 5 | 0 | 5 |
| P16_3 | 0 | 6 | 0 | | 0 | 0 | 0 | 6 |
| P16_4 | 0 | 6 | 0 | | 0 | 0 | 0 | 6 |
| P16_5 | 58 | 1 | 0 | | 0 | 0 | 111 | 170 |
| P16_6 | 6 | 0 | 0 | | 0 | 0 | 44 | 50 |
| P16_7 | 2 | 1 | 0 | | 0 | 0 | 6 | 9 |
| P16_8 | 5 | 0 | 0 | | 0 | 0 | 0 | 5 |
| P16_9 | 6 | 0 | 0 | | 0 | 0 | 15 | 21 |
| P16_10 | 1 | 1 | 1 | | 0 | 0 | 2 | 5 |
| P16_11 | 4 | 20 | 0 | | 0 | 0 | 58 | 82 |
| P16_12 | 6 | 26 | 0 | | 1 | 0 | 23 | 56 |
| P16_13 | 6 | 0 | 0 | | 0 | 0 | 21 | 27 |
| Total | 346 | 101 | 9 | | 4 | 5 | 710 | 1175 |

**LSIL**: low-grade squamous intraepithelial cervical lesion; **HSIL**: high-grade squamous intraepithelial cervical lesion; **Unspecified**: Unspecified Genital Site, hysteromyomas.

**NOTE**: The number represents the specific quantity of that source. This table summarizes only the recorded isolation sources and is not a complete representation of all data.
